# Through the *Cat-Map* Gateway: A Brief History of Cataract Genetics

**DOI:** 10.3390/genes15060785

**Published:** 2024-06-14

**Authors:** Alan Shiels

**Affiliations:** Department of Ophthalmology and Visual Sciences, Washington University School of Medicine, St. Louis, MO 63110, USA; alanshiels@outlook.com or shiels@wustl.edu

**Keywords:** lens, cataract(s), inherited, age-related, genes, mutations/variants

## Abstract

Clouding of the transparent eye lens, or cataract(s), is a leading cause of visual impairment that requires surgical replacement with a synthetic intraocular lens to effectively restore clear vision. Most frequently, cataract is acquired with aging as a multifactorial or complex trait. Cataract may also be inherited as a classic Mendelian trait—often with an early or pediatric onset—with or without other ocular and/or systemic features. Since the early 1990s, over 85 genes and loci have been genetically associated with inherited and/or age-related forms of cataract. While many of these underlying genes—including those for lens crystallins, connexins, and transcription factors—recapitulate signature features of lens development and differentiation, an increasing cohort of unpredicted genes, including those involved in cell-signaling, membrane remodeling, and autophagy, has emerged—providing new insights regarding lens homeostasis and aging. This review provides a brief history of gene discovery for inherited and age-related forms of cataract compiled in the *Cat-Map* database and highlights potential gene-based therapeutic approaches to delay, reverse, or even prevent cataract formation that may help to reduce the increasing demand for cataract surgery.

## 1. Introduction

**Figure 1:**
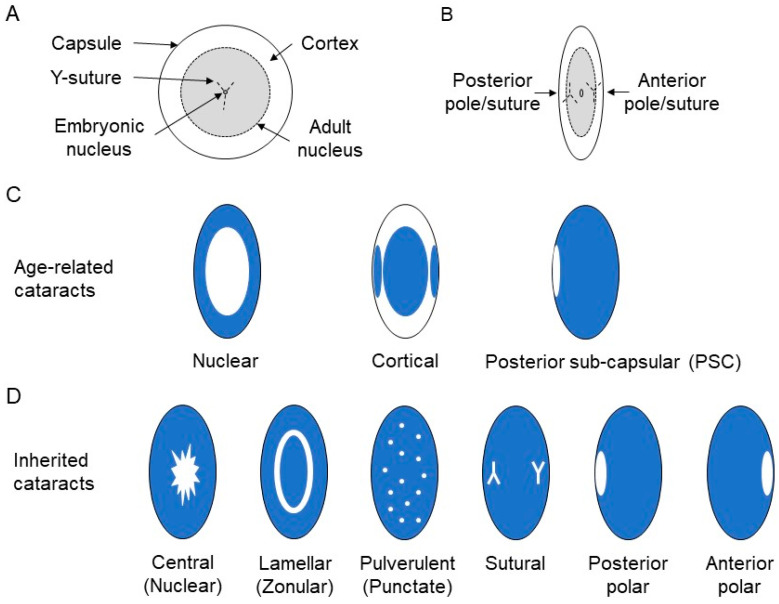
Schematic of lens and cataract morphology. Anterior (**A**) and equatorial (**B**) views of lens morphology related to cataract. In the newborn lens, the upright Y-suture at the anterior pole, the inverted Y-suture at the posterior pole, and the embryonic nucleus (0.3 mm diameter) are visible. The adult lens nucleus is similar in size to the newborn lens. Age-related cataract (**C**) presents as nuclear, cortical, or posterior sub-capsular opacities either separately or in combinations including total cataract. Inherited cataract (**D**) presents as highly heterogeneous opacities affecting single or multiple zones of the lens.

The ocular lens, along with the cornea, serves to focus images onto the photosensitive retina and plays an important role in refractive development [1]. The lens develops form surface (head) ectoderm under the control of integrated growth factor signaling and gene-regulatory networks to form an ellipsoidal cellular structure surrounded by a collagenous basement membrane or capsule that is suspended by the ciliary zonules in the anterior chamber of the eye [2,3,4,5]. The mature lens is composed of two cell phenotypes: an anterior monolayer of mitotically competent epithelial cells that terminally differentiate at the lens ‘equator’ region into concentric layers (growth-shells) of tightly packed, highly elongated, secondary fiber cells, which terminate in the so called ‘Y-sutures’ at the anterior and posterior poles and undergo programmed organelle loss to form the exquisitely patterned refractive mass of the lens [6,7,8,9,10]. The newborn lens (birth to 3 months) has an equatorial diameter of approximately 6.5 mm and an anterior–posterior or sagittal thickness of approximately 4 mm with a wet weight of approximately 65 mg increasing in size to around 9 mm × 4.5 mm weighing 250 mg in an 80-year-old adult [11]. In terms of lens morphology, the newborn lens is equivalent to the so-called nucleus of the adult lens and the surrounding adult cortex represents postnatal lens growth through accretion of lens fiber cells (Figure 1).

The glass-like transparency and refractive index of the living lens is largely derived from the short-range ordering in the spatial arrangement of long-lived, highly concentrated, cytoplasmic proteins known as crystallins [12].

Loss of lens transparency or cataract(s) is the clinical presentation of light scattering (diffraction, reflection, and absorbance) caused by protein aggregation and degeneration of the cellular microarchitecture within the lens resulting in blurred or obscured vision [12,13]. Cataract is a clinically important cause of visual impairment typically associated with aging and over 50% of Americans age 80 or older either have cataract or have undergone cataract surgery (https://www.nei.nih.gov/learn-about-eye-health/eye-conditions-and-diseases/cataracts). Despite recent advances in cataract surgery—which has been practiced in rudimentary forms such as ‘couching’ for over 2000 years [14]—senile or age-related cataract is the leading cause (approximately 45%) of global blindness afflicting an estimated 15 million adults (≥50 years of age) and the second leading cause (approximately 38%) of moderate and severe vision impairment (MSVI) affecting an estimated 78 million adults worldwide [15]. Clinical classification of age-related cataract using slit-lamp examination includes three main phenotypes—nuclear, cortical, and posterior sub-capsular cataract (PSC) in descending order of prevalence (Figure 1) [16]. These lens opacities may occur separately or in combination with varying degrees of severity or density—including total lens opacification—that can be graded using slit-lamp based clinical classification systems [17,18].

Beyond ageing, cataract may also be inherited as a classical Mendelian trait with autosomal dominant, autosomal recessive, or X-linked modes of inheritance—with or without other ocular and/or systemic defects—and usually with an early onset (0–40 years) [19,20,21]. While a report of Y-linked cataract is likely autosomal dominant [22], mitochondrial inheritance has been associated with congenital and age-related cataract [23,24]. Mendelian forms of cataract may present at birth (i.e., congenital), during infancy (infantile, 0–2 years), childhood (juvenile, 2–12 years), or adolescence (12–21 years), and beyond as pre-senile cataract before 40 years of age. While hereditary cataract is considered a rare disease (less than 4 cases/10,000 children), collectively, it accounts for approximately 22% of global congenital cataract [25]. Although ultrasound screening of an at-risk fetus can detect cataract in utero [26], congenital and infantile forms of cataract are typically detected in newborns by ophthalmoscope screening to visualize the so called ‘red reflex’ that ensures the reflected light from the retina is nor obscured by an opaque lens. Dense congenital and infantile cataracts that significantly reduce visual acuity during the critical period of visual development require early lens replacement surgery (under general anesthesia) with accurate refractive power correction within the first few months of life to avoid the risk amblyopia and loss of form vision [27,28]. While lens replacement surgery for congenital/infantile cataract provides the standard-of-care critical for a good visual outcome, it requires post-surgical monitoring for the development of glaucoma [29,30]. Recently, the introduction of lens regeneration surgery, which depends on in situ regrowth of the lens from endogenous lens epithelial ‘stem’ cells that are left largely intact following extraction of cataract material, has been proposed as an innovative surgical treatment for congenital/infantile cataract [31,32]. However, the efficacy of lens regeneration surgery as a treatment for cataract in humans remains controversial [33,34,35] and in cases of inherited cataract lens regeneration without genetic correction will result in cataract recurrence.

Slit-lamp imaging reveals that while inherited forms of cataract are usually bilateral and fairly symmetrical, their morphology is highly heterogeneous compared to age-related cataract with no standard classification system [36,37]. In one simplified system, four types of opacities have been identified namely, zonular, polar, total/mature, and membranous [38]. Zonular refers to a specific zone or region within the juvenile lens including central/nuclear affecting the lens core, lamellar affecting a layer of lens cells around the core, and sutural affecting the virtual Y-shaped suture branches that define the ends of fiber cells converging near the poles (Figure 1). Polar opacities affect the anterior and posterior poles of the lens including adjacent regions of the surrounding capsule. Membranous refers to the dense white membrane that results from fusion of the anterior and posterior capsule following resorption of the lens mass. Besides lens zone or location, the shape (e.g., aculeiform, coral-like, disc-like, polymorphic), density (e.g., dust-like/pulverulent, punctate), and even color (e.g., cerulean or blue-dot) of lens opacities are also highly variable. Moreover, inherited cataract may present as (1) an isolated or primary (non-syndromic) phenotype associated with mild anterior eye abnormalities (e.g., microcornea) [39] or even subtle metabolic defects (e.g., glucosuria, hyperferritinemia) or (2) as a secondary or variable feature of a diverse spectrum of genetic diseases (syndromic) including chromosome abnormalities (e.g., Down syndrome), inborn errors of metabolism (e.g., galactosemia-1), multisystem diseases (e.g., Lowe syndrome), and/or other ocular diseases (e.g., Stickler syndrome) [40,41,42,43].

While the original reports of familial cataract in Europe trace back to the late 1800s [44], gene discovery for inherited cataract was later pioneered by genetic linkage studies using various chromosome/genome-wide markers particularly microsatellite repeats and single nucleotide polymorphisms/variants (SNP/Vs) in extended pedigrees followed by positional cloning techniques and Sanger sequencing of linkage intervals. Indeed, an autosomal dominant zonular pulverulent (total nuclear) cataract (CZP1, CAE1)—segregating in a 6-generation English pedigree originally documented in 1909—became the first monogenic disease to be mapped to a specific human autosome following its linkage to the Duffy blood group gene (*FY*) that was later mapped to chromosome 1 in 1968 [45]. Subsequently, the first clue about candidate genes for hereditary cataract was provided by linkage of the autosomal dominant Coppock-like (discoid) cataract (CCL)—segregating in a 9-generation British pedigree first documented in 1910—to the γ-crystallin (*CRYG*) gene cluster on chromosome 2 [46]. More recently, the advent of massively-parallel or next-generation sequencing (NGS) of families and/or affected parent-child trios using candidate gene panels, whole exome sequencing (WES), and even whole genome sequencing (WGS) has greatly accelerated cataract gene discovery—largely replacing genetic linkage studies.

In contrast to Mendelian cataract, age-related cataract is a multifactorial or complex disease involving certain well studied environmental factors (e.g., UV-B exposure, cigarette smoking, and type-2 diabetes) and more recently emerging genetic factors including variable gene-environment and gene-gene interactions [21,47,48]. Early attempts to identify genetic risks for age-related cataract using twin and family studies have provided heritability estimates of approximately 35% for nuclear cataract and up to 53–58% for cortical cataract [49]. A genome-wide linkage scan using sibling pairs and microsatellite markers identified the first loci for age-related cortical cataract on chromosomes 6p (ARCC1) and 1p (ARCC2) along with suggestive evidence of linkage at five additional loci on chromosomes 1q31, 2p24, 2q11, 4q28, and 15q13 [50]. Such linkage scans have been surpassed by genome-wide association studies (GWAS) with SNP/Vs in large, multi-ethnic, case-control populations that identify multiple genetic associations to a complex or polygenic trait such as age-related cataract [51,52,53,54,55,56,57,58]. In addition, phenome-wide association studies (PheWAS) that complement GWAS by identifying multiple independent phenotypes associated with a single gene or variant (i.e., pleiotropy) are beginning to uncover complex genome-phenome relationships for cataract [55,59].

**Figure 2:**
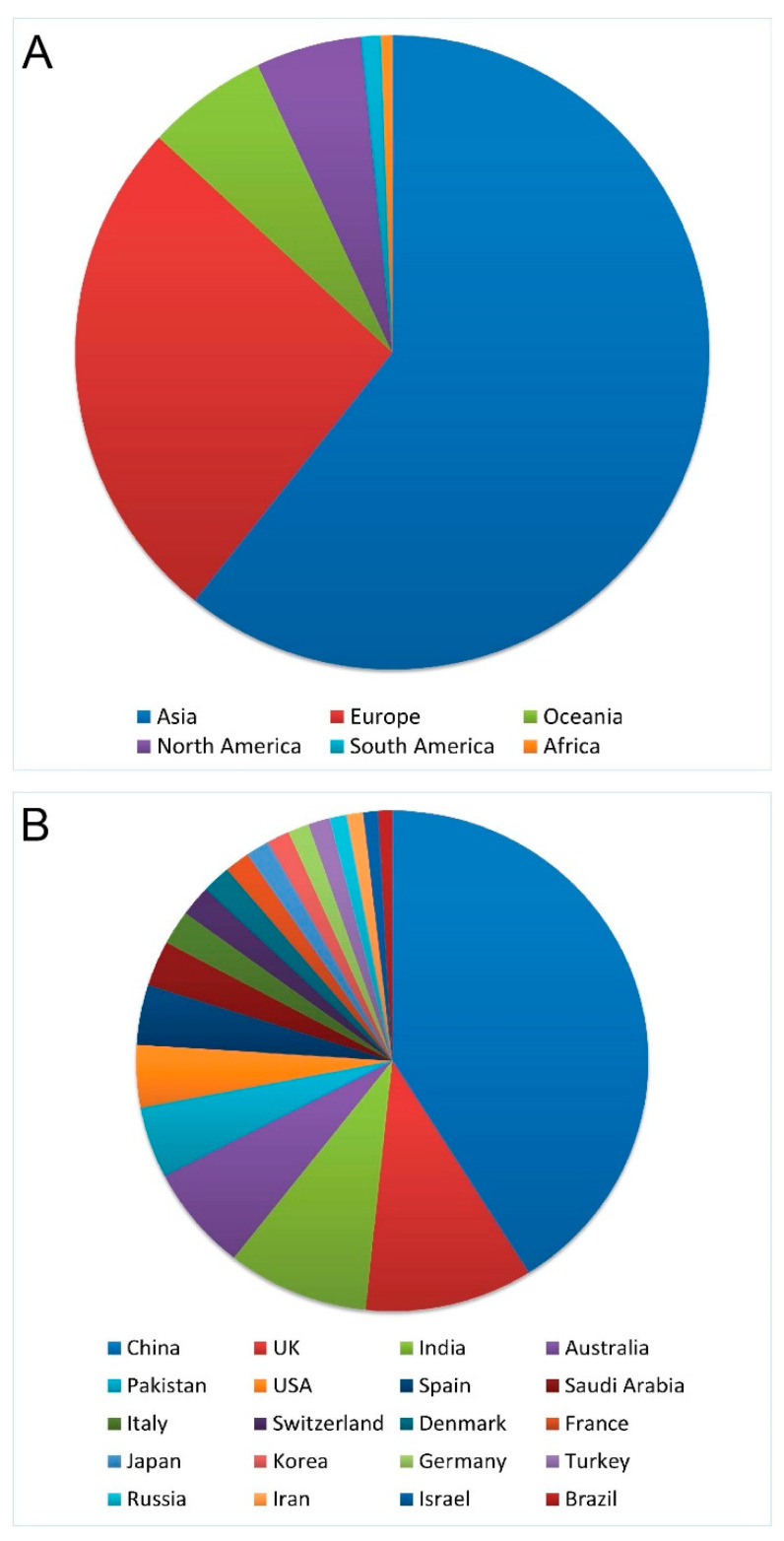
Schematic summarizing the geographic distribution of cataract genetics entries listed in *Cat-Map* by continent of origin (**A**) and by country of origin with 10 or more entries (**B**).

*Cat-Map:* Since the first reports of gene discovery for inherited cataract in the early 1990s, there has been a dramatic rise in genetic studies of cataract worldwide. In an attempt to keep track of and gain access to the genetic determinants of cataract, *Cat-Map* was compiled as a curated reference database and gene map for inherited and age-related forms of cataract [60] (https://cat-map.wustl.edu/, accessed on 12 June 2024). Keyword searches of PubMed (https://pubmed.ncbi.nlm.nih.gov/, accessed on 12 June 2024), Online Mendelian Inheritance in Man (OMIM, https://omim.org/, accessed on 12 June 2024), and other National Center for Biotechnology Information (NCBI, https://www.ncbi.nlm.nih.gov/, accessed on 12 June 2024) databases are used to identify relevant peer-reviewed literature reporting loci, genes, mutations/variants, mode-of-inheritance, geographic origin, cataract appearance, along with any co-existing ocular and/or systemic phenotypes. Genes and loci are listed in human chromosome order with genomic co-ordinates (Genome Reference Consortium Human Build 38/GRCh38) and syntenic mouse and other animal genes and models for cataract are appended. Mutations and variants are numbered using standard nomenclature recommendations [61] starting with the A of the translation start-codon (A_1_TG) and/or with reference sequence (rs) numbers. Currently, *Cat-Map* totals over 1850 entries including links to over 900 publications in the PubMed database originating from 6 continents and over 50 countries world-wide led by Asia and China, respectively, (Figure 2), along with links to over 450 human genes and loci in the NCBI Gene database (https://www.ncbi.nlm.nih.gov/gene/, accessed on 12 June 2024) that contribute to the cataract spectrum disorders. Most of these genes (>300) have been associated with syndromic forms of cataract that present as secondary and/or variable features of other primary diseases affecting the eye and/or multiple organ systems, which are underrepresented in *Cat-Map*. Such syndromic cataract genes and mutations are beyond the scope of this review and are covered in more detail elsewhere [40,41,42,43] including other gene databases (e.g., OMIM, https://omim.org/, accessed on 12 June 2024, GeneCards, https://www.genecards.org/, accessed on 12 June 2024).

This review chronicles gene discovery for inherited forms of isolated or primary cataract (±mild ocular and/or systemic abnormalities) and/or age-related cataract—largely based on the current update of the *Cat-Map* database (https://cat-map.wustl.edu/, accessed on 12 June 2024)—and highlights potential opportunities for gene-based cataract therapeutics. These cataract genes fall into three broad categories. (1) ‘Lens-signature’ genes specifically and/or abundantly expressed in the lens and/or eye that were predictably associated with cataract. (2) Genes widely expressed beyond the lens/eye that were unexpectedly associated with cataract. (3) Orphan loci for cataract without identified genes and lens expressed genes without cataract association (Table 1 and Table 2).

## 2. Lens-Signature Genes for Inherited and Age-Related Cataract

### 2.1. Genes for Lens Crystallins

It is perhaps fitting that one of the first identified genes for inherited cataract in humans and mice were members of the crystallin gene family (human gene symbol *CRY*, mouse gene symbol *Cry*, protein symbol CRY) that accounts for over 90% of the soluble protein in the lens and contributes significantly to the exquisite optical properties of the lens [9,12]. Following discovery of *Crybb2* and *Cryge* as the underlying genes for cataract in the *Philly* and eye-lens obsolescence (*Elo*) mouse mutants, respectively [62,63], it was proposed—albeit erroneously—that re-activation of the *CRYGE* pseudogene (*CRYGEP*) on the long-arm of chromosome 2 (2q) caused CCL in humans [64]. Subsequently, mutations in *CRYBB2* on 22q (cerulean opacities), *CRYAA* on 21q (zonular central nuclear, cortical, and posterior sub-capsular opacities), *CRYBA1* on 17q (zonular, sutural opacities) *CRYGC* on 2q (CCL opacities), and *CRYGD* on 2q (aculeiform, punctate opacities) were shown—in rapid succession—to underlie distinct types of inherited cataract in humans [65,66,67,68,69,70]. Currently, all 13 crystallin genes (*CRYAA/AB*, *CRYGA-GD/GS*, *CRYBB1-BB3/BA1/BA2/BA4*) spread across 7 human autosomes harbor over 440 single and recurrent mutations/variants associated with inherited and age-related forms of cataract (Table 1). Most crystallin gene mutations underlie autosomal dominant cataract and ‘crystallin cataracts’ can be associated with other ocular abnormalities (e.g., microcornea, microphthalmia, nystagmus, iris malformations), whereas several *CRYAB* mutations have been associated with different myopathies. *CRYAA*, *CRYGD*, and *CRYBB2* harbor over 50% of crystallin gene mutations including highly recurrent missense substitutions in *CRYAA* (p.R12C, p.R21W, p.R54C, p.R116C) and *CRYGD* (p.P24T), and a nonsense mutation in *CRYBB2* (p.Q155X). While variants in or near most crystallin genes have been tentatively associated with age-related nuclear cataract, only variants in *CRYAA* and *CRYAB* (11q)—which encode the small heat-shock proteins HSPB4 and HSPB5, respectively—exceed (*CRYAA*) or approach (*CRYAB*) genome-wide significance for association with age-related nuclear cataract in multi-ethnic Asian (*CRYAA*) or European (*CRYAB*) populations [52,54].

### 2.2. Genes for Lens Transmembrane and Beaded Filament Proteins

*GJA3 and GJA8:* Some 30 years after it was first linked to *FY* on chromosome 1, the mutation underlying CZP1/CAE1 and a second cataract mutation were discovered in the gene for gap-junction protein α-8 (*GJA8*) on the short-arm of chromosome 1 (1p) [71]. Soon after, *Gja8* was found to harbor the nuclear opacity (*Nop*) mutation in mice and the first mutations underlying autosomal dominant cataract linked to human chromosome 13q were found in *GJA3* [72,73]. *GJA3* and *GJA8* encode the two most abundant lens gap-junction proteins or connexins (Cx46 and Cx50, respectively) that function in lens intercellular communication including the fluid/ion micro-circulation system [9,74]. The mutation profiles of *GJA3* and *GJA8* rival those of the most mutated crystallin genes with over 220 mutations combined including several recurrent missense substitutions in *GJA3* (e.g., p.P59L) and *GJA8* (e.g., p.D47N). Both connexin genes predominantly underlie autosomal dominant cataract (mostly nuclear, zonular, pulverulent) and *GJA8* mutations tend to be associated with other eye defects including microcornea, microphthalmia, and nystagmus. Variants in both *GJA3* and *GJA8* have been suggestively associated with age-related cataract, but only one variant in *GJA3* had an ancestry-specific association with age-related nuclear cataract in Asians that approached genome-wide significance [54,75].

*MIP and LIM2:* Following the discovery of mutations in the mouse gene for major intrinsic protein (*Mip*) underlying cataract in the Fraser (*Cat^Fr^*) or Shriveled (*Svl*) mouse and the lens opacity (*Cat^lop^*) mouse mutants, originally described in the 1960s, the first mutations in the human counterpart gene (*MIP*) were discovered in autosomal dominant ‘polymorphic’ and lamellar cataracts linked to chromosome 12q [76,77]. Similarly, the first mutation in the mouse gene for lens intrinsic membrane protein 2 (*Lim2*) was identified in the Total opacity 3 (*To3*) mutant [78,79] and subsequently mutation of the human counterpart gene (*LIM2*) was found in autosomal recessive presenile cataract linked to chromosome 19q [80]. *MIP* encodes a member of the aquaporin gene family (AQP0) that is believed to facilitate lens water transport, cell-cell adhesion, and membrane-cytoskeleton interactions [81], whereas *LIM2* encodes a member of the peripheral myelin protein 22 (PMP-22)_Claudin superfamily (Pfam00822), and is believed to function in cell-cell adhesion, junction-formation, and fusion, in part, to generate the lens core syncytium [82,83]. Variants in or near *MIP* and *LIM2* have been nominally associated with age-related nuclear cataract but neither gene achieved genome-wide significance [54].

*BFSP1 and BFSP2*: Simultaneously, a recurrent codon deletion mutation and a missense mutation in the gene for beaded filament structural protein-2 (*BFSP2*) were first discovered in two American families with autosomal dominant congenital (nuclear, sutural, and stellate) cataract and juvenile-onset progressive cataract, respectively, linked to chromosome 3q [84,85]. Subsequently, a large deletion mutation in the gene for beaded filament structural protein 1 (*BFSP1*) was first discovered in an Indian family with autosomal recessive juvenile-onset cataract linked to chromosome 20q [86]. *BFSP1* and *BFSP2* encode lens-specific intermediate filament-like proteins that interact with complexes of CRYAA and CRYAB to form the distinctive beaded filament components of the lens cytoskeleton [87]. Variants in or near both *BFSP1* and *BFSP2* have been nominally associated with age-related nuclear cataract in Europeans but only *BFSP1* approached genome-wide significance [54].

### 2.3. Genes for Eye and/or Lens Transcription Factors

*PAX6*: Following discovery of mutations in the gene for paired-box 6 in mice (*Pax6*) and humans (*PAX6*) as causative for the *Small eye* (*Sey*) and aniridia, respectively [88,89], nonsense mutations in *PAX6* on chromosome 11p were found in a family trio with paternal congenital (lamellar and sub-capsular) cataract with late-onset corneal dystrophy, maternal aniridia, and syndromic anophthalmia with craniofacial and neurological defects in the compound-heterozygote proband—consistent with gene dosage effects [90]. *Pax6* was then identified as a master control gene for eye development [91,92] and, currently, over 250 *PAX6* mutations have been associated with anophthalmia, aniridia, microphthalmia, cataract and other defects in humans (Leiden Open Variation Database, https://databases.lovd.nl/shared/genes/PAX6, accessed on 12 June 2024). Subsequently, mutations in at least five other genes encoding transcription factors that operate downstream of PAX6 have been found to underlie cataract with or without other (anterior) ocular and occasional neurological abnormalities (Table 1).

*PITX3:* A highly recurrent duplication mutation (c.640_656dup17bp) in the human gene for paired-like homeodomain transcription factor 3 (*PITX3*) on chromosome 10q was first discovered in families with autosomal dominant cataract and anterior segment mesenchymal dysgenesis (ASMD) [93]. Subsequently, a deletion mutation in the promotor region of *Pitx3* was found to cause aphakia (ak) and small eyes in a mouse mutant first described in the 1968 [94]. Several other single and recurrent *PITX3* mutations have been reported in autosomal dominant posterior polar/subcapsular cataract that tends to be accompanied by ASMD or other eye defects (e.g., microphthalmia, Peter’s anomaly)—consistent with a key transcription factor role in lens and anterior eye development.

*FOXE3*: Following the discovery of two missense mutations in the gene for forkhead box E3 (*Foxe3*) underlying the autosomal recessive dysgenetic lens (dyl) phenotype in mice first reported in 1979 [95,96], an insertion mutation was first discovered the human counterpart gene (*FOXE3*) on chromosome 1p in a family with autosomal recessive cataracts and anterior segment ocular dysgenesis (ASOD) [97]. FOXE3 function is required for closure and separation of the lens vesicle and for survival and proliferation of the lens epithelium—suggesting that the accompanying ASOD resulted from lens defects [95,96].

*MAF*: Soon after mutations of the musculoaponeurotic fibrosarcoma proto-oncogene (*MAF*) on chromosome 16q were first discovered in families with autosomal dominant congenital (pulverulent) cataract and other ocular defects including microcornea and iris coloboma, a *Maf* mutation was found to underlie the dominant Opaque flecks in lens (Ofl) cataract phenotype in mice [98,99]. Subsequently, multiple mutations in *MAF* have been found in cataract with or without other ocular abnormalities or as part of the multisystem Aymé-Gripp syndrome [100]. MAF is a basic region leucine zipper (bZIP) transcription factor of the activator protein-1 (AP-1) superfamily that is expressed during lens development to regulate crystallin gene expression and several mutations have been found to alter its DNA-binding activity—highlighting the role of the lens in anterior eye development.

*HSF4:* Missense mutations in the gene for heat-shock transcription factor-4 (*HSF4*) on chromosome 16q were first discovered in three Chinese families with autosomal dominant lamellar cataract and in a multi-generation Danish pedigree segregating the historically important Marner (zonular stellate and anterior polar) cataract first described in 1949 [101]. Subsequently, a transposon insertion mutation in *Hsf4* was found to underlie the autosomal recessive lens opacity 11 (lop 11) and allelic lens disrupter-1 (ldis1) phenotypes in mice [102]. HSF4 functions in the transcriptional control of heat shock proteins in the lens (e.g., CRYAB) and, unlike other ocular transcription factors, *HSF4* mutations underlie isolated forms of cataract with autosomal dominant inheritance caused by missense mutations in the conserved α-helical DNA-binding domain and autosomal recessive inheritance caused by null mutations outside the DNA-binding domain.

*SOX2-OT:* Nonsense mutations in the gene for sex determining region Y (SRY)-box transcription factor 2 (*SOX2*) on chromosome 3q were first discovered in cases of anophthalmia—consistent with a critical role in eye development [103]. Recently, using multi-ethnic GWAS meta-analyses, variants in the gene for *SOX2* overlapping transcript (*SOX-OT*) have been reproducibly associated with age-related (nuclear) cataract at a level exceeding genome-wide significance [54,55]. *SOX2-OT* encodes a conserved long (>3 kb) non-coding RNA (lncRNA) believed to regulate expression of *SOX2* itself, which lies within an intron of *SOX2-OT*, suggesting a novel gene dysregulation mechanism for cataract formation.

## 3. Widely Expressed Genes for Inherited and Age-Related Cataract

Beyond, the usual suspect genes and ocular transcription factor genes outlined above, a functionally diverse cohort of widely expressed genes—including protein-coding and non-coding genes—continues to be associated with inherited cataract with or without ocular/systemic abnormalities and in some cases with age-related cataract. Collectively, this emerging group of ‘extra-lenticular’ genes (currently >30) greatly expands the genetic heterogeneity of cataract and provides new insights regarding lens development, homeostasis, and aging (Table 1).

*GALK1:* Homozygous mutations in the galactokinase 1 gene (*GALK1*) on chromosome 17q were first discovered in families with autosomal recessive galactokinase 1 deficiency with cataract or galactosemia-type 2 [104]. GALK1 catalyzes the first step of galactose metabolism and loss of enzyme function results in galactitol accumulation in the lens causing osmotic cataract that can be ameliorated by a lactose-free diet or enzyme replacement therapy. Subsequently, the so-called ‘Osaka’ variant (p.A198V) in *GALK1*, which inhibits enzyme activity, has been associated with age-related cortical cataract in a Japanese population but not in an Italian/European population [105,106].

*FTL:* Non-coding mutations in the iron response element (IRE) located in the 5′-region of the gene for ferritin-light chain gene (*FTL*) on chromosome 19q were first shown to underlie autosomal dominant cataract with hyperferritinemia (in the absence of iron overload anemia). Since then, over 100 single and recurrent mutations in the IRE, a short, conserved stem-and-loop structure, have been shown to drive overexpression of FTL to crystallin-like levels in the lens [107,108,109].

*GCNT2:* Mutations in the gene for glucosaminyl (N-acetyl) transferase 2 (*GCNT2*) on chromosome 6p, which encodes the I-branching enzyme responsible for the adult i blood-group phenotype, underlie autosomal recessive cataract in Japanese but not Europeans—due to differential splicing of *GCNT2* transcripts in red-cells versus lens cells and the different population-specific location of mutations within *GCNT2* [110,111].

*NHS*: Mutations in the gene for the X-linked Nance-Horan/cataract-dental syndrome (*NHS*) on chromosome Xp, which encodes an actin remodeling regulator, may present as isolated cataract particularly in female carriers of the disease [112].

*TMEM114:* 3 Mutations in the gene for transmembrane protein 114 (*TMEM114*) on chromosome 16p, including a familial t(16;22) balanced translocation, were first linked with autosomal dominant congenital (lamellar) cataract [113]. TMEM114 is a glycosylated member of the PMP22_Claudin family and was formerly identified as a claudin component of tight junctions—but has subsequently been assigned to the more distantly related voltage dependent calcium channel γ subunits (CACNGs) [114].

*CHMP4B:* Several mutations the gene for charged multivesicular body protein 4B (*CHMP4B*) on chromosome 20q were first linked with autosomal dominant (posterior polar/subcapsular) cataract in American and Japanese families [115]. CHMP4B is a core component of the endosomal sorting complex required for transport III (ESCRT-III) membrane remodeling and scission machinery and may fulfill a gap-junction specific role in the lens by forming complexes with GJA8/Cx50 [116].

*EPHA2:* Concurrently, the first mutations in the human gene for ephrin type-A receptor 2 (*EPHA2*) on chromosome 1p were found to underlie autosomal dominant cataract and several germline variants (but not somatic variants) were associated with age-related cortical cataract mapped to the ARCC2 locus [117,118,119,120]. Variants in the genes for ephrinA1 (*EFNA1*) and ephrinA5 (*EFNA5),* which encode known ligands of EPHA2, have also been associated with age-related cataract but only *EFNA1* reached genome-wide significance [55,121]. *EPHA2* and *EFNA1* are two of 18 genes involved in notochord development and EPHA2 plays a specialized role in lens cell pattern formation [55,122].

*SLC16A12:* Mutations and variants in the genes for several members of the solute carrier (SLC) gene family have been associated with cataract. A nonsense mutation in the gene for monocarboxylate transporter 12 or creatine transporter 2 (*SLC16A12*) on chromosome 10q, was first shown to underlie autosomal dominant juvenile cataract with or without microcornea and renal glucosuria [123]. Subsequently, variants in or near *SLC16A12*, *SLC7A8* (14q), which codes for the large neutral amino acids transporter small subunit 2, and *SLC24A3* (20p), which codes for the sodium/potassium/calcium exchanger 3, have also been associated with age-related cataract but only *SLC24A3* achieved genome-wide significance [55,124,125].

*VIM:* A handful of mutations in the gene for the ubiquitous cytoskeletal protein vimentin (*VIM*) on chromosome 10p have been associated with autosomal dominant cataract [126].

*FYCO1:* Multiple mutations, including nonsense, frameshift and missense, in the gene for FYVE (Fab1p, YOTB, Vac1p, and EEA1) and coiled-coil domain containing protein 1 (*FYCO1*) on chromosome 3p were first linked with autosomal recessive congenital cataract segregating in Pakistani and Israeli families [127]. FYCO1 functions as an autophagy adaptor in the translocation of autophagosomes and plays a role in both basal autophagy and elevated autophagy in the lens required for the programmed elimination of lens fiber cell organelles from the light path [128].

*TDRD7:* Loss-of-function mutations in a gene for Tudor domain containing protein 7 (*TDRD7*) on chromosome 9q were first linked with autosomal recessive cataract and glaucoma [129]. Mice lacking *Tdrd7* develop cataract, glaucoma, and arrested spermatogenesis [129]. TDRD7 is a component of RNA granules and functions as an RNA-binding protein required for post-transcriptional control of mRNA transcripts that are critical for lens development and retinal ganglion cell function [129].

*mtDNA:* Variants in the mitochondrial (mt) genes for NADH dehydrogenase subunit 1 (*ND1*) and *ND5* were first associated with congenital (central/total, zonular) cataract of maternal origin in families of Indian ancestry [23]. ND1 and ND5 are components of the respiratory Complex I that initiates the electron transport chain of mitochondrial oxidative phosphorylation and variants affecting these proteins may lead to oxidative stress in the lens triggering cataract formation [23].

*AGK:* Soon after loss-of-function mutations in the gene for acylglycerol kinase (*AGK*) on chromosome 7q were first reported in Sengers syndrome (98% of cases with congenital cataract), a splice-site mutation in *AGK*, resulting in exon deletion and protein truncation, was linked with isolated autosomal recessive congenital cataract [130]. AGK is a mitochondrial membrane lipid kinase that catalyzes the formation of phosphatidic and lysophosphatidic acids, which are involved in cell signaling suggesting that cataract may result from unbalanced lipid composition and/or abnormal signaling during lens development [130].

*CYP51A1:* A novel missense variant in the gene for cytochrome P450, family 51, subfamily A, member 1 (*CYP51A1*) on chromosome 7q was first associated with autosomal recessive congenital (lamellar/cortical) cataract in a consanguineous Arab family [131]. CYP51A1 functions as lanosterol 14-α-demethylase in the cholesterol biosynthesis pathway suggesting that a deficiency of this enzyme may cause abnormal sterol metabolism leading to cataract [131].

*AKR1E2:* A splice variant in the gene for aldo-ketoreductase family 1, member 2 (*AKR1E2*) on chromosome 10p was first associated with autosomal recessive congenital (complete) cataract in a consanguineous Arab family [131]. AKR1E2 catalyzes the conversion of 1,5-anhydro-D-fructose to 1,5-anhydro-D-glucitol suggesting that deficiency of this enzyme may lead to the accumulation of sorbitol or other polyols that cause osmotic cataract [131].

*RNLS:* A truncation (deletion/insertion) variant in the gene for renalase (*RNLS*) on chromosome 10q was first identified in an Arab family with autosomal recessive congenital cataract [131]. RNLS functions as a secreted flavin adenine dinucleotide (FAD)-dependent monoamine oxidase involved in catecholamine metabolism and may play a role in mitigating mitochondrial dysfunction [132].

*WFS1:* A heterozygous missense mutation in the gene for autosomal recessive Wolfram syndrome (*WFS1*) on chromosome 4p, which includes diabetes insipidus, diabetes mellitus, optic atrophy, and deafness (DIDMOAD) with or without congenital cataract, was first found in an Irish pedigree segregating isolated autosomal dominant congenital nuclear cataract [133]. WFS1 (Wolframin) functions as an endoplasmic reticulum (ER)-resident transmembrane glycoprotein and may lead to cataract through impaired membrane targeting, protein processing/secretion, and/or ER calcium homeostasis during lens development [133].

*UNC45B:* A missense mutation in the gene encoding un-coordinated 45 myosin chaperone B (*UNC45B*) on chromosome 17q was first linked with autosomal dominant juvenile cataract in Danish pedigree [134]. UNC45B functions as a myosin-specific co-chaperone suggesting that juvenile cataract may result from defective assembly of non-muscle myosin during lens development and fiber cell differentiation [134].

*TRPM3:* Mutation of the gene for transient receptor potential (TRP) cation channel subfamily M (melastatin), member 3 (*TRPM3*) on chromosome 9q was first linked with autosomal dominant early onset, progressive cataract with or without glaucoma in an African American family [135]. TRPM3 functions as a steroid-activated, heat-sensitive calcium channel and appears to play a key role in calcium dynamics during lens development, aging, and cataract formation [136,137]. SNP/Vs in TRPM3 have also been nominally associated with age-related nuclear cataract and incipient senile cataract [54,59].

*COL4A1:* A missense mutation in the gene coding for collagen α-1(IV) chain (*COL4A1*) on chromosome 13q was first associated with autosomal dominant congenital nuclear cataract in a Chinese family [138]. COL4A1 is an abundant component of the lens capsule that is secreted by the lens epithelium and the cataract associated mutation may result in protein misfolding in the ER and activation of the unfolded protein response [138].

*SIPA1L3:* A *de novo* translocation breakpoint and a heterozygous missense mutation in the gene for signal-induced proliferation-associated 1 like 3 (*SIPA1L3*) on chromosome 19q were first associated with presumptive autosomal dominant cataract and anterior segment abnormalities and a homozygous nonsense mutation in *SIPA1L3* was found in isolated autosomal recessive cataract [139,140]. SIPA1L3 is a GTPase-activating protein for small G-proteins of the Rap (Ras-related) family suggesting that cataract may result from defects in epithelial cell morphogenesis, polarity, and cytoskeletal organization during lens development [139,140].

*LSS*: Homozygous missense mutations in the gene for lanosterol synthase (*LSS*) on chromosome 21q were first found in two families segregating autosomal recessive, congenital, total cataract and subsequently compound heterozygous mutations in *LSS* were found in cases of nuclear cataract with hypotrichosis [141,142,143]. A variant in *LSS* was reported to confer susceptibility to age-related nuclear cataract in a Chinese population, whereas no statistically significant variants in *LSS* were associated with age-related cataract in Europeans [144,145]. LSS catalyzes the conversion of (S)-2,3 oxidosqualene to lanosterol—a rate-limiting cyclization step in the biosynthesis of cholesterol, steroid hormones, and vitamin D—and loss of LSS function may elicit cataract formation by reducing the ability of lanosterol to prevent protein aggregation in the lens [146].

*LONP1:* Homozygous mutations in the gene for mitochondrial lon peptidase 1 (*LONP1*) on 19p were first associated with autosomal recessive congenital (central/nuclear) cataract in consanguineous Arab families with or without CODAS (cerebral, ocular, dental, auricular, skeletal) syndrome [147,148]. LONP1 (aka, lon protease homolog, mitochondrial) belongs to the Lon family of ATP-dependent proteases and functions in the degradation of misfolded or damaged proteins/polypeptides in the mitochondrial matrix [147,148].

*WDR87:* The first homozygous missense mutation in the gene for WD repeat-domain containing protein-87 (*WDR87*) on chromosome 19q was associated with autosomal recessive congenital (complete/total white) cataract in an Arab family [147]. While WDR87 participates in spermatozoa tail assembly it’s function in the lens is unknown.

*LEMD2:* A homozygous missense mutation in the gene for LEM (LAP2, emerin, LAN1) domain-containing protein 2 (*LEMD2*) on chromosome 6p was first linked with the autosomal recessive, Hutterite-type, juvenile-onset, cataract initially reported in 1985 [149]. In addition, this Hutterite-type cataract was associated with sudden cardiac death. *LEMD2* codes for a transmembrane protein that localizes to the inner nuclear membrane, interacts with the nuclear lamina, and is involved in nuclear membrane organization—suggesting that juvenile cataract may result from dysfunction of the nuclear envelope in the developing lens [149].

*RRAGA:* Mutations in the gene for Ras-related GTP binding protein A (*RRAGA*) on chromosome 9p were first associated with autosomal dominant (sub-capsular and nuclear) cataract [150]. RRAGA functions as a key regulator of the mechanistic target of rapamycin complex 1 (mTORC1) signaling cascade suggesting that *RRAGA* mutations may lead to deleterious effects on the autophagy pathway [150].

*PANK4:* A non-coding (intron 4) variant in the gene for (pseudo/inactive) pantothenate kinase 4 (*PANK4*) on chromosome 1p was first linked with autosomal dominant congenital posterior cataract in a Chinese family [151]. PANK4 lacks the catalytic domain present in PANK1-3 that is required for the phosphorylation of the vitamin pantothenate to 4′-phosphopantothenate as the first step in the biosynthesis of co-enzyme A (CoA). While the function of PANK4 in the lens is unclear, mice lacking *Pank4* develop cataract and the human variant, which may elicit a transcript splicing error, reduced PANK4 expression in the blood of affected family members. *PANK4* was also mooted as a candidate gene for the Volkmann type cataract (see below) [151].

*DNMBP:* Bi-allelic loss-of-function mutations in the gene for dynamin binding protein (*DNMBP*) on chromosome 10q were first linked with autosomal recessive bilateral infantile- or early childhood-onset cataract in three consanguineous Pakistani families [152]. Further, a variant in *DNMBP* has been associated at genome-wide significance with age-related cataract in women but not men suggesting that sex-specific genetic effects can influence cataract development [55]. DNMBP is a member of the guanine nucleotide exchange factor family, which regulates the configuration of cell (tight) junctions and acts as a scaffold protein that interacts with dynamin to regulate the actin cytoskeleton—suggesting that loss of DNMBP function may impair cytoskeleton dynamics in the lens leading to cataract formation [152].

*RP1-140A9.1:* A splice-site variant (rs763295804G/C) in the long non-coding (lnc)RNA gene *RP1-140A9.1* on chromosome 1p has been shown to co-segregate with autosomal dominant congenital (central, zonular, sutural) cataract, Volkmann type (CCV) with incomplete penetrance in a multi-generation Danish family first linked to chromosome 1p in 1995 [153]. *RP1-140A9.1* is a primate-specific, two-exon gene with two alternative transcripts—one of which was expressed in fetal eye and lens epithelial cells—that may function as an antisense RNA in gene regulation or as an microRNA ‘sponge’. The associated variant affects the consensus donor splice-site and is predicted to cause intron skipping to generate an unprocessed RNA transcript—providing the first example of a point-mutation in an lncRNA gene underlying autosomal dominant cataract. However, a regulatory effect on expression of the gene for guanine nucleotide-binding (G) protein subunit β 1(*GNB1*), located downstream, could not be excluded [153].

*AQP5:* A missense mutation in the gene for aquaporin 5 (*AQP5*) on chromosome 12q was first discovered in a Chinese family with autosomal dominant congenital (sutural, nuclear) cataract [154]. AQP5 functions as a dynamic water channel believed to contribute to the lens ion/fluid microcirculation system and a mouse model of human AQP5 deficiency developed mild age-related lens opacification [81,154].

*PIKFYVE:* A likely pathogenic missense mutation in the gene for phosphoinositide kinase, FYVE-type zinc finger containing (*PIKFYVE*) on chromosome 2q has been found in a 4-generation Chinese Korean family with autosomal dominant cataract (nuclear pulverulent, sutural, cortical punctate) cataract—in the absence of fleck corneal dystrophy [155]. PIKFYVE functions as a 1-phosphatidylinositol 3-phosphate 5-kinase that regulates endomembrane homeostasis and a zebrafish model of PIKYVE deficiency, but not transgenic overexpression of the PIKYVE missense variant, mimicked human cataract—consistent with a haploinsufficiency mechanism rather than a dominant-negative inhibition of PIKFYVE kinase activity [155].

*VUS:* Finally, in addition to mutations in known genes for cataract, variants of unknown significance (VUS) in the genes for ATP binding cassette subfamily B member 6 (*ABCB6*), acetyl-CoA carboxylase α/1 (*ACACA*), charged multivesicular body protein 4A (*CHMP4A*), chloride intracellular channel protein 5 (*CLIC5*), heat shock protein family E (Hsp10) member 1 (*HSPE1*), outer dense fiber of sperm tails 1 (*ODF1*), and transient receptor potential cation channel subfamily melastatin, member 1 (*TRPM1*) have been associated with non-syndromic (likely autosomal dominant), congenital cataract (including nuclear, lamellar, posterior subcapsular/polar, and pulverulent opacities) in a cohort of Spanish families—and these await further validation as cataract-causing mutations [156,157].

## 4. GWAS Continue to Expand the List of Widely Expressed Genes for Age-Related Cataract

Beyond pioneering family-based studies of inherited cataract, more recent population-based genome-wide studies of age-related cataract continue to expand the genetic diversity of cataract by identifying variants in or near an increasing number of associated risk loci/genes (>50), some of which have been replicated at genome-wide significance levels (*p* = 5 × 10^−8^) using multi-ethnic GWAS meta-analyses.

In a GWAS of type 2 diabetes (T2D) patients, 15 SNPs located near several novel genes (*PPARD*, *CCDC102A*, *GBA3*, *NEDD9*, *GABRR1/2*, *RPS6KA2*, *TAC1*, *GALNTL1* and *KIAA1671*) on six chromosomes were putatively associated with diabetic cataract in a Taiwanese population [51], whereas, a variant in the gene for calcium voltage-gated channel subunit α-1 C (*CACNA1C*) was associated with diabetic cataract in a Scottish T2D cohort [158].

Variants in *CRYAA* (see above) and the gene coding for voltage-gated potassium channel subunit β-1 (*KCNAB1*) have been associated with age-related nuclear cataract in Asians but only *CRYAA* association was replicated in Europeans—consistent with ethnicity-specific effects [52,54]. In addition, variants in *SOX-OT* (see above) and in the genes for transmembrane serine protease 5 (*TMPRSS5*), the long intergenic non-protein coding RNA 1412 (*LINC01412*), BRD4-interacting chromatin-remodeling complex-associated protein/glioma tumor suppressor candidate region gene 1 protein (*BICRA/GLTSCR1*), and copper metabolism domain containing 1 (*COMMD1*) were associated with age-related nuclear cataract at GWAS significance in Europeans and Asians [54].

Beyond nuclear genes, a mitochondrial genome-wide association study (miWAS) has identified a mitochondrial variant (rs41323649/MitoG228A) that was associated with a 5-fold increased risk of age-related cataract in a US Latino population [24].

At least 54 GWAS significant loci/genes (37 novel), including replication of SOX-OT, SLC24A3, and 15 other genes (CDKN2C, DPM3-KRTCAP2, ADCK3, IGFBP3-TNS3, ZNF800, CDKN2B-DMRTA1, BAMBI-LINC01517, ODF3-BET1L, 5′-LOC338694, OCA2, WWP2, MIR2117HG, NPLOC4, 3′-METRNL, and JAG1), have been associated with cataract in multi-ethnic populations including African American, Asian, European, and Hispanic cohorts [53,55]. Further, sex-stratified analyses, identified several genes significantly associated with cataract in females (CASP7, DPNB-CPN1) but not males and several genes associated with cataract in males (QKI, SEMA4D, RBFOX1, JAG1) but not females—consistent with sex-specific effects influencing cataract onset [55]. Approximately 80% of the identified genes exhibited differential expression during lens development and pathway analysis revealed robust enrichment for the notochord development gene-set, which includes EPHA2 and EFNA1 [55].

A GWAS of age-related cataract in a Guatemalan (Mayan) cohort identified 33 significantly associated SNPs of which ~25% were intergenic and the rest were located in 13 genes including seven protein-coding genes (*ACSL1*, *CFAP74*, *CTNNA3*, *FRMD4B*, *NAALADL2*, *PRCP*, and *ZNF423*), two non-coding RNAs (*LINC00968*, *PENK-AS1*), and four uncharacterized loci (LOC105377670, LOC101927668, LOC107984170, LOC105369844) [56]. Pathway analysis further suggested that four of the cataract-associated genes were involved in DNA replication (*ZNF423*), signal transduction (*PRCP*), metabolism (*ACSL1*), and the immune system (*CTNNA3*).

Finally, using nationwide electronic health records, a biallelic missense variant in the gene for caspase 7 (*CASP7*) on chromosome 10q has been strongly associated with first diagnosis of adult-onset cataract (~63 years-of-age) in a biobank of the Finnish population, whereas mono-allelic (heterozygous) carriers had a later onset—suggestive of a recessive phenotype [57]. *CASP7* has also been shown to be significantly associated with age-related cataract in females [55]. While CASP7 is one of the executioner caspases in the apoptosis pathway that is expressed in the lens, it does not appear to activate during the programmed organelle loss characteristic of lens fiber cells [159].

## 5. Orphan Loci and Genes for Cataract

Despite advances in high-throughput sequencing techniques, over 10 loci for inherited cataract have been mapped to chromosome regions without identification of the underlying genes (Table 2). These include at least seven loci for autosomal dominant cataract on chromosomes 2p (CTRCT29, CTRCT27), 12q (CTRCT37), 14q (CTRCT32), 15q (CTRCT25), 17p (CTRCT24), 17q (CTRCT7), two loci for autosomal recessive cataract on 9q (CTRCT26) and 19q (CTRCT35), one locus for X-linked cataract, along with a locus for age-related cortical cataract on chromosomes 1p (CTRCT6) and 6p (CTRCT28). Conversely, several genes known to be important in lens development and differentiation have yet to be directly associated with cataract. These include genes for galectin-related inter-fiber protein (*GRIFIN*, 7p), which binds α-crystallin [160,161], lengsin (*LGSN*, 6p) a pseudo-glutamine synthase which may function as a cytoskeletal chaperone [162], along with deoxyribonuclease-2-β (*DNASE2B*, 1p), BCL2 interacting protein 3 like (*BNIP3L*, 8p) and phospholipase A and acyl transferase 3 (*PLAAT3*, 11q), which are involved in the programmed degradation of lens fiber cell nuclei and other organelles in animal models [10,163,164] (Table 2). Focused studies of these ‘orphan’ cataract loci and lens genes will provide new insights into lens and cataract development.

## 6. Gene-Based Therapeutics for Cataract

While lens replacement surgery for cataract, pioneered by NHL Ridley in 1949–1950 [165], is a highly effective treatment for cataract, its limited access in certain developing countries and increasing demand and associated healthcare cost in developed countries have prompted studies to find alternative interventions to delay, reverse, or even prevent cataract onset. Besides testing various antioxidant compounds, aspirin-like drugs, aldose reductase inhibitors, and histone acetyl transferase inhibitors for treating experimental forms of age-related and/or diabetic cataracts in animal models [146,166,167,168], genetic studies of human cataract have begun to suggest novel therapeutic approaches to cataract including small-molecule eye drops and potentially gene-editing.

*UPR:* Mouse models of human autosomal dominant cataract mimicking mutations in *GJA8* [169], *CRYAA* [170], *CRYBA3* [171], and *MIP* [172] have suggested that overactivation of the unfolded protein response (UPR) leads to induction of apoptosis/cell-death pathways. Similarly, activation of the UPR via distinct pathways has been found in age-related and congenital cataracts [173] suggesting that the UPR may provide a common therapeutic target(s) for multiple types of cataracts.

*Oxysterols:* The discovery of mutations in *LSS* underlying autosomal recessive cataract in humans has prompted studies in using lanosterol, an amphipathic compound present at high levels in the lens, to reverse protein aggregation in cataract formation in vitro and in dogs [141]. Similarly, in mouse models mimicking human *CRYAA*- and *CRYAB*-related cataract the oxysterol chaperone compound 25-hydroxycholesterol was found to reduce protein aggregation and partially restore lens transparency [174]. In vitro modeling of *CRYGD*-(p.P24T)- and *CRYBB2*-(p.Q155X)-related cataract has suggested that lanosterol can attenuate protein aggregation and opacification in lentoid bodies regenerated from patient-specific induced pluripotent stem cells derived from urine (UiPSCs) [175]. Despite these promising results however, the anti-cataractogenic properties of lanosterol and other oxysterol compounds have been disputed. In vitro studies of human lenses with age-related nuclear cataract and chemically induced cataract in rat lenses, revealed that neither lanosterol immersion nor lanosterol liposomes dissolved aggregated lens proteins or restored lens transparency [176,177]. In the Shumiya cataract rat (SCR-C) model, which inherits homozygous mutations in *Lss* and the gene for farnesyl diphosphate farnesyl transferase 1 (*Fdft1*), which both function in the cholesterol biosynthesis pathway, intravitreal injection or a topical gel of lanosterol nanoparticles were reported to delay degeneration of lens structure but failed to prevent cataract onset [178,179].

*Autophagy:* In a zebrafish model of a human *GJA8* ablation, loss of Cx50 gap-junction function was found to decrease (macro)autophagy and cause cataracts, which could be relieved by the autophagy stimulator rapamycin [180]. Similarly, in a zebrafish model of human PIKFYVE loss-of-function, aberrant lens vacuolation caused by the accumulation of amphisomes—formed by the fusion of autophagosomes and late endosomes—was largely alleviated by treatment with the V-ATPase inhibitor bafilomycin A1 (Baf-A1) [155]. Several other autophagy-related genes underlying inherited forms of primary cataract including *CHMP4B*, *FYCO1*, *TDRD7*, and *RAGA*, as well as those underlying syndromic forms of cataract including *TBC1D20* (TBC1 domain family, member 20), *EPG5* (ectopic P-granules autophagy protein 5), and *VPS4* (vacuole protein sorting 4a) support the role of autophagy in maintaining lens differentiation and homeostasis [128] and suggest that autophagy may provide therapeutic targets for delaying or preventing cataract formation.

*Gene therapy:* Gene-editing techniques, including the clustered regularly interspaced short palindromic repeats (CRISPR)-CRISPR-associated nuclease 9 (Cas9) system, have also been tested as a potential therapeutic approach for certain types of inherited cataract [181]. In a mouse model of human *CRYGC*-related autosomal dominant cataract, gene-editing using CRISPR-Cas9 oligonucleotide reagents injected at the zygote stage resulted in the rescue of cataract in approximately 30% of subsequent offspring [182]. CRISPR-Cas9 gene-editing in vitro of spermatogonial stem-cells derived from a mouse model of human *CRYGC*-related cataract has been shown to correct the genetic defect and rescue the cataract phenotype in subsequent offspring with 100% efficiency [183]. Conversely, rabbit models of human *GJA8*-related and *CRYAA*-related inherited cataract have been generated using CRISPR-Cas9 gene-editing at the zygote stage to inform studies of cataract pathogenesis and potential pharmacotherapy [184,185]. In addition, an efficient method for evaluating candidate genes and variants of unknown significance implicated in human cataract has been developed in zebrafish embryos using CRISPR-Cas9 ribonucleoprotein (RNP) complexes [186]. These CRISPR-based model systems provide validating evidence for cataract causation, rather than mere association, and may pave the way for gene therapy approaches, potentially coupled with lens regeneration surgery, for certain forms of inherited cataract.

## 7. Summary and Outlook

Induction of dominant cataract in the first-generation offspring of male mice (spermatogonia) exposed to experimental doses of ionizing radiation (e.g., γ-rays,) and chemical mutagens (e.g., ethylnitrosourea/ENU) has long been used as a ‘dosimeter’ to estimate genetic mutation risk in humans [187]. By analogy, the broad clinical spectrum of inherited (syndromic and non-syndromic) and age-related forms of cataract in humans, points to the sensitivity of the lens to diverse genetic mutations/variations involving over 450 genes across the nuclear (22 autosomes and X-chromosome) and mitochondrial genomes. In cases of idiopathic pediatric cataract, where other causes including intrauterine infection (e.g., Rubella), drug exposure (e.g., corticosteroids), or ionizing radiation have been excluded and a genetic etiology is suspected, relatively rapid molecular diagnosis may be achieved in up to 50–90% of cases [42,188] using commercially available cataract gene panels (e.g., blueprintgenetics.com) and high-throughput sequencing techniques (e.g., whole exome) to provide enhanced genetic counseling for patients and their extended families.

Approximately 20 lens-signature genes harboring over 590 single and recurrent mutations/variants account for most forms of isolated or primary cataract with autosomal dominant inheritance (Table 1). While the crystallin genes were predicted to account for most if not all inherited forms of primary cataract, seven other genes (*GJA3/8*, *MIP*, *LIM2*, *BFSP1/2*, *HSF4*) now account for a significant proportion (~44%) of autosomal dominant cataract. However, there is little genotype-phenotype correlation making clinical classification based on cataract appearance challenging. Overall, however, these cataract genes recapitulate much of what is known about lens development and differentiation.

Beyond the lens, over 25 widely expressed and functionally diverse genes (coding and non-coding) have been associated with inherited forms of primary cataract and in some cases with syndromic and age-related forms of cataract—consistent with pleiotropic effects (Table 1). Although mutations in widely expressed genes (e.g., *FTL*, *EPHA2*) mostly underlie autosomal dominant forms of cataract, studies of consanguineous families have identified a number of diverse candidate genes for autosomal recessive cataract including *GCNT2*, *FYCO1*, *LSS*, *DNMBP* and *PIKFYVE* (Table 1)—providing new insights into lens biology and pathology. However, cataract gene discovery remains incomplete—with at least 25 orphan loci lacking causative genes (Table 2).

Predictions that genes underlying inherited congenital cataract might be informative about the genetics of age-related cataract have been supported at least in some cases [189]. Variants in many genes underlying inherited forms of cataract (e.g., *BFSP1*, *LIM2*, *MIP*, and *CHMP4B*) have been shown to exhibit tentative association with age-related nuclear cataract [54]. So far, however, only variants in *CRYAA* and *SOX-OT* have been replicated at genome-wide significance in multi-ethnic populations with age-related cataract [52,54,55]. In addition, variants in or near over 50 widely expressed and functionally diverse genes have been significantly associated with age-related cataract [55]. Some of these GWAS variants exhibit ethnic and/or sex-specific influences on age-related cataract formation and pathway analysis has revealed significant enrichment of genes involved in notochord development (e.g., *EPHA2*) [55].

While functional studies of several candidate genes for human cataract including *CRYAA, CRYGC, GJA8*, and *MIP* using animal models have provided compelling evidence for cataract causation, functional studies of GWAS variants are more challenging even if the candidate gene in question underlies inherited cataract. For example, a mutant mouse model homozygous for a rare coding variant in *EPHA2* (p.R722Q) associated with age-related cataract in humans did not develop cataract but did exhibit subtle defects in lens cell patterning and suture formation [122]—consistent with a weak phenotypic effect. Moreover, functional studies of GWAS significant variants—especially those that lie in introns or other non-coding/intergenic regions near candidate genes—will likely require multi-variant testing in animal models in order to detect polygenic mechanisms that increase susceptibility to or protection from age-related cataract. Other GWAS-based approaches such as PheWAS have utilized variants associated with age-related cataract in order to identify genetic correlations with other diseases/phenotypes (i.e., pleiotropic analyses) [55,59]. Besides their association with other lens/eye disorders, several variants associated with age-related cataract were found to be located in genes that were significantly associated with hypertension, diabetes, and anthropometric traits (e.g., body fat mass) suggesting genetic links between age-related cataract and metabolic syndrome. Yet other cataract-associated variants were also associated with skin and eye pigmentation phenotypes [55]. Similarly, a Mendelian randomization approach, which uses genetic variants to determine the likelihood that observed risk factors are causative for a disease, has revealed that genetically determined primary open-angle glaucoma and myopic refractive error were significantly associated with risk of age-related cataract [58]. Future studies focused on human genome-phenome relationships will provide new information about the pathogenetic mechanisms, including gene-gene and gene-environment interactions, underlying age-related cataract and potentially other ocular and/or systemic diseases or conditions.

Overall, despite the burgeoning genetic complexity of cataract intriguing genetic links between genes involved in eye development, early-onset cataract, age-related cataract, and certain other ocular and extraocular diseases are beginning to emerge that may contribute to the design of novel therapeutic interventions and/or public health strategies to alleviate the increasing demand for cataract surgery.

## Figures and Tables

**Table 1:** Genes for inherited and age-related cataract.

No.	Locus	Gene	Cataract Phenotype	Phenotype MIM No.	Gene MIM No.	*Cat-Map* AD	*Cat-Map* AR	*Cat-Map* Sporadic/?	*Cat-Map* Complex	*Cat-Map* Total
1	1pter-p36.13	*RP1-140A9.1*	CTRCT8, multiple types (cataract, congenital, Volkman-type, CCV)	115665	?	2	0	0	0	2
2	1p36.32	*PANK4*	CTRCT49, (posterior)	619593	606162	1	0	0	0	1
3	1p36.13	*EPHA2*	CTRCT6, multiple types (age-related cortical cataract, ARCC2)	116600	176946	24	7	3	14	48
4	1p33	*FOXE3*	CTRCT34, multiple types (±microcornea, CATC3)	612968	601094	5	13	8	0	26
5	1q21.1	*GJA8*	CTRCT1, multiple types (±microcornea, CZP1, CAE1)	116200	600897	113	13	16	6	146
6	2q33.3	*CRYGB*	CTRCT39, multiple types	615188	123670	2	0	1	0	3
7	2q33.3	*CRYGA*	multiple types/nuclear	?	123660	3	1	0	0	4
8	2q33.3	*CRYGC*	CTRCT2, multiple types (±microcornea, CCL)	604307	123680	44	0	9	0	53
9	2q33.3	*CRYGD*	CTRCT4, multiple types (±microcornea)	115700	123690	70	0	8	0	78
10	2q34	*PIKFYVE*	nuclear pulverulent, sutural, cortical punctate	?	609414	0	2	0	0	2
11	2q35	*CRYBA2*	CTRCT42	115900	600836	5	0	1	0	6
12	3p21.31	*FYCO1*	CTRCT18	610019	607182	0	31	4	0	35
13	3q22.1	*BFSP2*	CTRCT12; multiple types (±myopia)	611597	603212	11	2	1	0	13
14	3q26.33	*SOX2-OT*	age-related (nuclear) cataract	?	616338	0	0	0	2	2
15	3q27.3	*CRYGS*	CTRCT20; multiple types	116100	123730	11	0	1	0	12
16	4p16.1	*WFS1*	CTRCT41 (Wolfram syndrome/DIDMOAD)	116400	606201	6	2	4		12
17	6p24	*GCNT2*	CTRCT13 (+ adult i blood-group phenotype)	116700	600429	0	16	0	0	16
18	6p21.31	*LEMD2*	CTRCT46, juvenile onset	212500	616312	1	1	0	0	2
19	7q21.2	*CYP51A1*	lamellar/cortical?	?	601637	1	2	0	0	3
20	7q34	*AGK*	CTRCT38 (Sengers syndrome)	614691	610345	0	1	0	0	1
21	9p13.2	*RRAGA*	nuclear, posterior subcapsular	?	612194	2	0	0	0	2
22	9q21.12-q21.13	*TRPM3*	CTRCT50; ± glaucoma	620253	608961	2	0	0	0	2
23	9q22.33	*TDRD7*	CTRCT36	613887	611258	1	6	3	1	11
24	10p15.1	*AKR1E2*	congenital, complete?	?	617451	0	1	0	0	1
25	10p13	*VIM*	CTRCT30; pulverulent	116300	193060	3	0	2	0	5
26	10q23.31	*RNLS*	congenital?	?	609360	0	1	0	0	1
27	10q23.31	*SLC16A12*	CTRCT47, juvenile, with microcornea (±glucosuria)	61208	611910	1	0	2	8	11
28	10q24.2	*DNMBP*	CTRCT48	618415	611282		3	1	1	5
29	10q24.32	*PITX3*	CTRCT11; multiple types (microphthalmia, neurodevelopmental abnormalities included)	610623	602669	34	2	0	0	36
30	11p13	*PAX6*	cataract with late-onset corneal dystrophy	106210	607108	1	0	0	0	1
31	11q22.3	*CRYAB*	CTRCT16; multiple types (±myopathy, multiple types)	613763	123590	14	5	6	3	28
32	12q13.12	*AQP5*	sutural, nuclear	600231	600442	2	0	0	0	2
33	12q13.3	*MIP*	CTRCT15; multiple types	615274	154050	42	1	5	4	52
34	13q12.1	*GJA3*	CTRCT14; multiple types	601885	121015	66	3	10	3	82
35	13q34	*COL4A1*	non-syndromic congenital nuclear	?	120130	2	0	0	0	2
36	16p13.2	*TMEM114*	lamellar, central, polar, sutural	?	611579	3	0	0	0	3
37	16q21	*HSF4*	CTRCT5; multiple types	116800	602438	22	11	4	1	38
38	16q22-q23	*MAF*	CTRCT21; multiple types (±microcornea)	610202	177075	14	0	16	0	29
39	17q11.2	*CRYBA1*	CTRCT10; multiple types	600881	123610	42	1	9	0	52
40	17q12	*UNC45B*	CTRCT43	616279	611220	1	0	0	0	1
41	17q25	*GALK1*	Galactokinase deficiency + cataract (Galactosemia II, GALAC2)	230200	604313	1	32	2	1	36
42	19p13.2	*LONP1*	central/nuclear	600373	605490	0	2	6	0	8
43	19q13.13	*WDR87*	complete/total white	?	620274	0	1	0	0	1
44	19q13.1-13.2	*SIPAIL3*	CTRCT45	616851	616655	1	2	2	0	5
45	19q13.3	*FTL* (IRE)	Hyperferritinemia ± cataract	600886	134790	78	1	32	1	112
46	19q13.41	*LIM2*	CTRCT19	615277	154045	8	3	0	3	14
47	20p12.1	*BFSP1*	CTRCT33; cortical	611391	603307	4	2	4	1	11
48	20q11.22	*CHMP4B*	CTRCT31; multiple types	605387	610897	6	0	0	1	7
49	21q22.3	*CRYAA*	CTRCT9; multiple types (±microcornea)	604219	123580	51	7	15	11	80
50	21q22.3	*LSS*	CTRCT44	616509	600909	0	3	0	1	4
51	22q11.23	*CRYBB2*	CTRCT3; multiple types (±microcornea)	601547	123620	50	5	17	2	74
52	22q11.23	*CRYBB3*	CTRCT22; multiple types	609741	123630	9	3	5	0	16
53	22q12.1	*CRYBB1*	CTRCT17; multiple types	611544	6009291	18	7	7	0	32
54	22q12.1	*CRYBA4*	CTRCT23	610425	123631	9	1	3	1	14
55	Xp22.13	*NHS*	CTRCT40 (Nance-Horan/cataract-dental syndrome)	302200	300457	0	0	6	0	75

Total *Cat-Map* entries include single and recurrent mutations/variants associated with autosomal dominant (AD), autosomal recessive (AR), X-linked (X-L), sporadic or unknown (?) cataract inheritance and age-related (complex) cataract.

**Table 2:** Orphan loci and genes for cataract.

No.	Locus	Cataract Phenotype	Inheritance	OMIM No.
1	1p31.1-p22.3	*DNASE2B*		608057
2	1q25-q31	Nuclear	AD	
3	2p24-pter	CTRCT29 (coralliform)	AD	115800
4	2p12	CTRCT27 (nuclear progressive, CCNP)	AD	607304
5	2q33	Hexagonal, nuclear, cortical riders	AD	
6	2q37-qter	Posterior polar	AD	
7	2q37.3	Posterior polar	de novo	
8	3p26.2 [t(3;4)(p26.2;p15)]	Total	AD	
9	3p22.3 [t(3;5)(p22.3;p15.1)]	Embryonal nuclear, sutural, punctate	AD	
10	3q22.3-q25.2	Coronary	AD	
11	3q26.1-3q27.2	Congenital, bilateral	AR	
12	6p12-q12	CTRCT28, (cataract age-related cortical 1, ARCC1)	Complex	609026
13	6q12	*LGSN*		611470
14	7p22.3	*GRIFIN*		619187
15	7q21.11-q31.1	Nuclear	AR	
16	8p23.2-p21.3	Laminar, nuclear	AR	
17	9q13-q22	CTRCT26, multiple types (cataract autosomal recessive early-onset pulverulent, CAAR, CTPL1)	AR	605749
18	11q12.3-q13.1	*PLAAT3*		613867
19	12q24.2-q24.3	CTRCT37 (cataract congenital cerulean type 5, CCA5)	AD	614422
20	14q22-q23	CTRCT32, multiple types (cataract posterior polar 5, CTPP5; cataract anterior polar 1, CTAA1)	AD	115650
21	15q21-q22	CTRCT25 (cataract central saccular/pouch-like, sutural opacities, CCSSO)	AD	605728
22	16p13.3 [t(2;16)(p22.3;p13.3)]	Microphthalmia isolated with cataract 1 (MCOPCT1), cataract congenital with microphthalmia (CATM)	AD	156850
23	17p13	CTRCT24 (cataract anterior polar 2, CTAA2)	AD	601202
24	17q24	CTRCT7 (cataract congenital cerulean type 1, CCA1)	AD	115660
25	19q13	CTRCT35 (cataract congenital nuclear 1, CATCN1)	AR	609376
26	19q13	Cortical	AD	
27	19q13-qter	Nuclear	AD	
28	20p11.23-p12.2	Progressive congenital zonular nuclear cataract (ADPCZNC)	AD	
29	20p11.23-p12.1	Infantile, total	AD	
30	Xq24	Lamellar, nuclear, sutural, white dots	XL	

## Data Availability

The data presented in the study are available in *Cat-Map* at https://cat-map.wustl.edu [60].
